# Establishment of a 3D Co-Culture System Using OFT Cell Line and Effect of Hormone Supplementation on Germline Gene Expression in Olive Flounder (*Paralichthys olivaceus*)

**DOI:** 10.3390/cells15171572

**Published:** 2026-08-29

**Authors:** Jae Hoon Choi, Hee Jeong Kong, Jung-Ha Kang

**Affiliations:** 1 Biotechnology Research Division, National Institute of Fisheries Science, Busan 46083, Republic of Korea; genetics@korea.kr; 2 Genetics and Breeding Research Center, National Institute of Fisheries Science, Geoje 53334, Republic of Korea; heejkong@korea.kr

**Keywords:** 3D culture, testicular aggregates, in vitro model, olive flounder

## Abstract

In vitro 3D cell culture systems provide a physiologically relevant microenvironment that mimics native tissue architecture. However, establishing functional 3D testicular aggregates in teleosts remains challenging. In this study, we established a novel 3D co-culture aggregate system for olive flounder (*Paralichthys olivaceus*) primary testicular cells by incorporating the established olive flounder testicular cell (OFT) line. Primary testicular cells alone failed to form 3D aggregates within the first 24 h in ultra-low attachment plates. However, co-culturing with OFT cells rapidly initiated 3D aggregate assembly within 24 h. Furthermore, supplementation with a cocktail of sex hormones and growth factors maintained high cell viability without cytotoxic effects. Notably, quantitative real-time PCR analysis revealed that hormone and growth factor supplementation significantly upregulated the transcript levels of both *plzf* (2.15-fold) and *scp3* (2.34-fold). These results demonstrate that the OFT cell line is essential for facilitating the assembly and incorporation of primary testicular cells into 3D aggregates, while sex hormones and growth factors promote spermatogonial stem cell maintenance and meiotic progression within the aggregates. Overall, this 3D co-culture platform serves as a valuable in vitro tool for fish spermatogonial stem cell research and reproductive biotechnology in aquaculture.

## 1. Introduction

Germline stem cells (GSCs) transmit their genetic information to the next generation through complex interactions with somatic cells [1,2]. Utilizing this unique capability, surrogate broodstock technology, which involves producing donor-derived gametes in recipient organisms by transplanting donor GSCs, has emerged as a promising tool in aquaculture for the production of superior offspring, preservation of valuable genetic resources, and enhancement of aquaculture productivity [3]. However, the limited number of GSCs available from donor individuals presents a significant challenge to the practical application of this technology [4]. Thus, establishing efficient in vitro culture systems for GSCs is essential.

Several studies have reported the in vitro culture of GSCs from various teleost species. The first male GSC line, SG3, was established from medaka (*Oryzias latipes*) and maintained for over two years without feeder cells while retaining the expression of germ cell-specific markers [5]. Other studies have reported similar achievements in species such as swamp eel (*Monopterus albus*) [6], Chinese hook snout carp (*Opsariichthys bidens*) [7], orange-spotted grouper (*Epinephelus coioides*) [8], and tiger puffer fish (*Takifugu rubripes*) [9]. Nevertheless, the successful production of fertile gametes directly from GSCs cultured without somatic support remains largely unattained. In contrast, studies using co-cultures of GSCs with testicular somatic cells have demonstrated that maintaining close germ cell–somatic cell interactions is critical for supporting GSC survival, proliferation, and differentiation, as seen in zebrafish (*Danio rerio*) [10,11] and rainbow trout (*Oncorhynchus mykiss*) [4].

The olive flounder (*Paralichthys olivaceus*) is a commercially important aquaculture species in East Asia, especially in Korea, Japan, and China [12]. Various efforts, including selective breeding [13], triploid induction [14,15], and genome editing using CRISPR/Cas9 [16], have been made to improve its aquaculture traits. However, in vitro culture systems for *P*. *olivaceus* GSCs have not yet been developed.

In a previous study, an olive flounder testicular somatic cell (OFT) line was used to serve as a supporting cell source for male *P*. *olivaceus* GSCs culture [17]. However, conventional two-dimensional (2D) culture systems often fail to culture spermatogonial stem cells over 7 days. To better mimic the in vivo niche and improve GSC support, 3D co-culture models have been proposed as a promising strategy.

Therefore, in this study, we aimed to induce 3D testicular aggregates by co-culturing primary dispersed testicular cells from *P*. *olivaceus* with the established OFT cell line. Furthermore, we evaluated the effects of sex hormones and growth factors on the morphology, viability, and expression of germ cell markers within the aggregates. This approach is expected to provide fundamental insights for the development of functional in vitro GSC culture systems in *P*. *olivaceus* and contribute to advancing reproductive biotechnology in aquaculture species.

## 2. Materials and Methods

### 2.1. Experimental Fish and Ethical Approval

Nine of *P*. *olivaceus* fish, 1 year old (non-spawning season), with an average body weight of 364.2 ± 99.0 g and total length of 30.1 ± 2.6 cm were provided by and maintained at the National Institute of Fisheries Science (NIFS, Busan, Republic of Korea). The fish were kept in 1-ton flow-through tanks under a controlled photoperiod, with the water temperature strictly maintained at 19 ± 0.3 °C. They were fed a commercial pellet diet (Daebong LS Co., Ltd., Jeju, Republic of Korea) twice a day. All experimental procedures involving animals adhered to the Animal Protection Act of the Ministry of Agriculture, Food and Rural Affairs, Republic of Korea, and were officially approved by the Institutional Animal Care and Use Committee (IACUC) of NIFS (Approval No. 2025-NIFS-IACUC-20). For experiments, to ensure a sufficient and uniform starting population of cells, testes were collected and pooled from three individuals to create one independent pool. A total of three independent pools was generated from the nine fish.

### 2.2. Preparation of the Olive Flounder Testicular Cell Line

The olive flounder testicular cell (OFT) line used in this study was previously established and characterized by our group [17]. Briefly, OFT cells were cultured in modified ESM2 (mESM2), consisting of high-glucose Dulbecco’s modified Eagle’s medium (DMEM; Gibco, Grand Island, NY, USA) supplemented with 20 mM HEPES (Sigma-Aldrich, St. Louis, MO, USA), 2 mM L-Glutamine (Gibco), 1 mM non-essential amino acids (Gibco), 1 mM sodium pyruvate (Gibco), 2 nM sodium selenite (Sigma-Aldrich), 100 μM 2-mercaptoethanol (Gibco), 100 U/mL penicillin–streptomycin (Gibco), 15% FBS (Gibco), 10 ng/mL bFGF, 1% fish serum, and 50 μg/mL *P. olivaceus* embryo extract prior to their incorporation into the 3D aggregate culture system.

### 2.3. Isolation of Primary Testicular Cells

To isolate primary testicular cells, the procedure was adapted from our previously established protocols [17]. *P. olivaceus* individuals were deeply anesthetized using seawater supplemented with 0.1% (*v*/*v*) 2-phenoxyethanol (Sigma-Aldrich) for 10 min. Testes were sterilely excised and rinsed three times with 1 × Dulbecco’s phosphate-buffered saline (DPBS) to eliminate cellular debris and blood. The tissues were partitioned into 0.5 g fragments and finely minced in 100 mm culture dishes (Corning, Corning, NY, USA) using a No. 20 surgical blade (Paragon, Sheffield, UK). For enzymatic digestion, the minced tissues were incubated in 0.25% (*v*/*v*) trypsin-EDTA (Gibco) for 2 h at 20 °C, with intermittent gentle pipetting performed every 10 min. Trypsinization was terminated by adding an equivalent volume of mESM2. The cell suspension was sequentially passed through 100 μm (Falcon, Durham, NC, USA) and 40 μm strainers (Falcon) to filter out undigested tissue remnants. Finally, the filtrate was centrifuged at 400× *g* for 10 min to harvest the primary cell pellet for downstream applications.

### 2.4. Induction of 3D Co-Cultured Testicular Aggregates

To evaluate the effects of OFT on the formation of aggregates, 5 × 10^5^ cells of isolated testicular cells and 5 × 10^5^ OFT cells were seeded into ultra-low attachment 96-well plates (ULA; Corning). They were cultured for 14 days with culture medium exchanged every 2 days. For control, 1 × 10^6^ cells of OFT cells were seeded into ULA plates. mESM2 was used for culturing aggregates, but mESM2 supplemented with 10 ng/mL of 11-ketotestosterone (11-KT), 10 ng/mL of 17β-estradiol (E_2_), 10 ng/mL of 17α, 20β-dihydroxy-4-pregnen-3-one (DHP), 10 ng/mL of human chorionic gonadotropin (hCG), 10 ng/mL of epidermal growth factor (EGF), and 10 ng/mL of insulin-like growth factor 1 (IGF-1) was also used to evaluate the effect of those supplements on the morphology, viability, and levels of germ cell-specific genes.

### 2.5. Assessment of Aggregate Morphology and Cell Viability

The effects of sex hormones on the structural characteristics and survival of the 3D testicular aggregates were assessed simultaneously. Morphological changes were routinely observed and captured using an inverted microscope (Zeiss, Jena, Germany). To determine the cell viability within aggregates, aggregates collected on day 14 were dispersed by 0.25% (*v*/*v*) trypsin-EDTA. Subsequently, the viability was measured by trypan blue (Gibco) staining. The spatial distribution of live and dead cells within day 14 aggregates was investigated using the Live/Dead Viability/Cytotoxicity Kit (Molecular probes, Eugene, OR, USA). Aggregates were stained according to the manufacturer’s protocol. As a negative control, harvested testicular aggregate was immersed in 70% (*v*/*v*) ethanol for 1 h.

### 2.6. Quantitative Analysis of Germline Marker Genes

Total RNA was extracted from the day 14 aggregates using TRIzol reagent (Thermo Fisher Scientific, Waltham, MA, USA). A total of 100 ng of extracted RNA was reverse transcribed into cDNA using SuperScript^TM^ IV VILO^TM^ Master mix with ezDNase (Invitrogen, Vilnius, Lithuania). The synthesized cDNA from each culture condition of aggregates was used for quantitative real-time polymerase chain reaction (qRT-PCR) analysis using the LightCycler^®^ 480 SYBR Green Master Mix (Roche Diagnostics, Mannheim, Germany). The reaction conditions were as follows: initial denaturation at 94 °C for 3 min, followed by 40 amplification cycles (denaturation at 94 °C for 30 s, annealing at 60 °C for 30 s, and elongation at 72 °C for 30 s). Primer sequences for each target gene were newly designed, and their amplification efficiencies were verified to be comparable prior to quantification. Primer sequences are listed in Table 1. Relative gene expression was determined using the 2^−ΔΔCt^ method. The ΔCt was calculated by subtracting the Ct value of the internal reference gene (*18s rRNA*) from the Ct of the target gene, and the ΔΔCt was obtained by comparing the ΔCt values between experimental and control samples [18]. *18s rRNA* was selected as the sole reference gene because its expression remained highly stable across all hormone treatment conditions in our preliminary tests.

### 2.7. Statistical Analysis

Data are represented as means ± standard deviation (SD) of three independent biological replicates (n = 3, representing independent culture experiments from different batches of primary cells). Statistical differences in cell viability and relative gene expression levels were analyzed by a *t*-test using SPSS software (version 19.0; SPSS Inc., Chicago, IL, USA) to compare the differences between the un-supplemented control (Group3) and the hormone-supplemented group (Group 4). Significant differences were considered when the *p* value was ≤0.05.

## 3. Results

### 3.1. Effects of Sex Hormones, Growth Factors, and OFT Cells on Testicular Aggregates

To evaluate the effects of sex hormones, growth factors, and OFT cell co-culture on 3D aggregate morphology, primary testicular cells were cultured in ULA plates across four experimental groups:

1. Group 1: 1 × 10^6^ primary testicular cells alone.
2. Group 2: Group 1 treated with sex hormones and growth factors.
3. Group 3: 5 × 10^5^ primary testicular cells co-cultured with 5 × 10^5^ OFT cells.
4. Group 4: Group 3 treated with sex hormones and growth factors.

**Figure 1:**
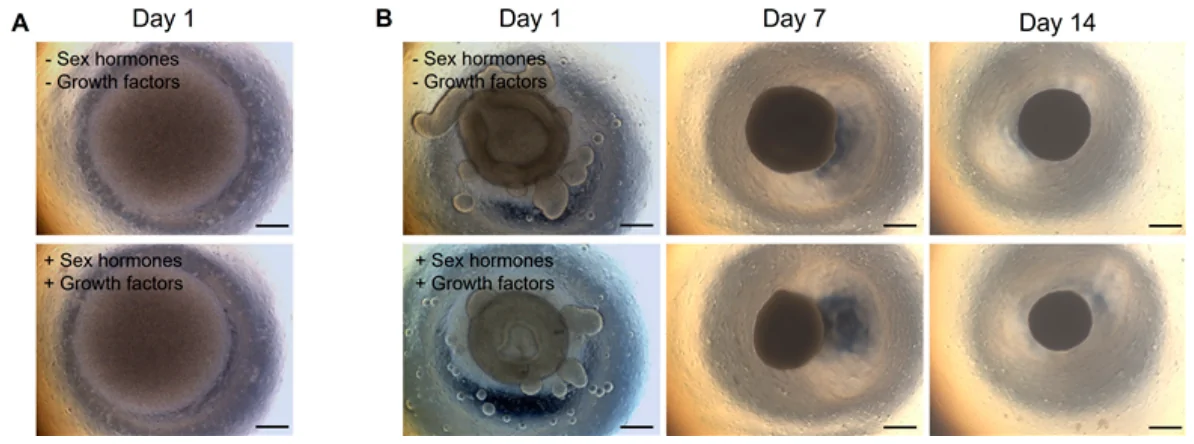
Effects of sex hormones and growth factors, and OFT cell co-culture on 3D testicular aggregate formation in olive flounder (*Paralichthys olivaceus*). (**A**) Representative morphological images of primary testicular cells cultured in ULA on day 1, with or without sex hormones and growth factors. (**B**) Representative images (Day 1, 7, and 14) of primary testicular cells co-cultured with OFT cells under the identical treatment conditions described in (**A**). The images illustrate the progressive assembly from multiple initial cell clusters on day 1 into a single, compact spherical 3D aggregate over 14-day culture period. Scale bar = 400 µm.

On day 1 of culture, no aggregate formation was observed in either Group 1 or Group 2, regardless of hormone or growth factor supplementation (Figure 1A). Since primary cells alone failed to form any aggregates within the first 24 h, long-term morphological progression was not observed. In contrast, testicular aggregate formation was initiated on day 1 in both Group 3 and Group 4, independent of hormone or growth factor treatment (Figure 1B). Initially, multiple distinct cell clusters were formed on day 1. Over extended culture periods, these clusters aggregated into a single, compact spherical aggregate per well. These results suggest that while sex hormone and growth factor supplementation is not critical for initial aggregate formation, the inclusion of the OFT cell line significantly promotes and accelerates 3D aggregate assembly (Figure 1B). Consequently, Group 3 and Group 4 were selected for subsequent downstream experiments. Since OFT cells cultured alone also exhibit an intrinsic capacity to form aggregates, their primary role in this co-culture system is likely not driving the aggregation of primary germ cells into the 3D structures. However, as the exact germ cell content and spatial distribution were not directly verified in this study, this incorporation remains an interpretation that requires future histological validation.

### 3.2. Effects of Sex Hormones and Growth Factors on the Cell Viability of 3D Aggregates

**Figure 2:**
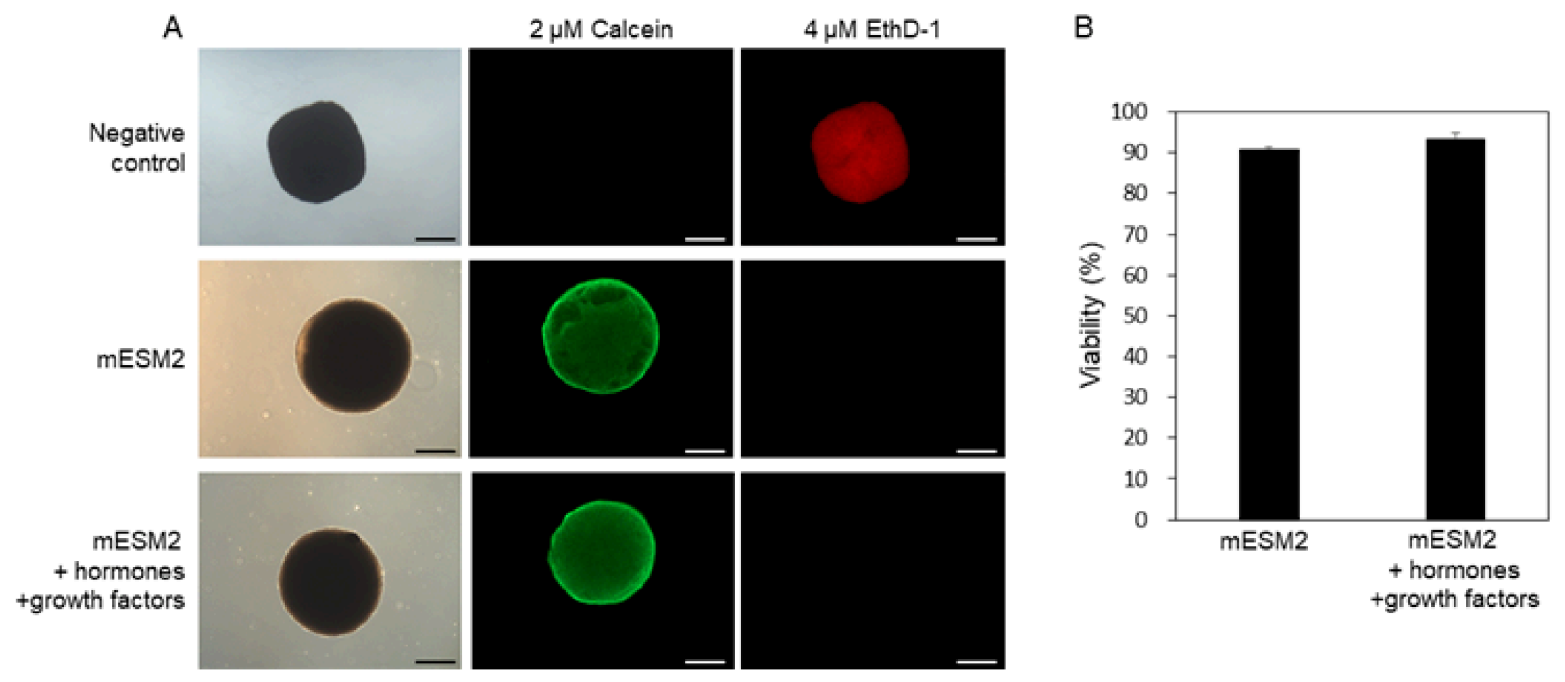
Effects of sex hormones and growth factors on the cell viability of 3D co-cultured testicular aggregates. (**A**) Representative fluorescence microscopy images following live/dead cell staining. The negative control was treated with 70% ethanol for 1 h. Viable cells were stained with 2 µM calcein-AM (green), and dead cells were stained with 4 µM ethidium homodimer-1 (EthD-1, red). Scale bar = 400 µm. (**B**) Quantitative comparison of cell viability within testicular aggregates cultured under different supplementation conditions, determined via trypan blue staining. Data are presented as mean ± SD (n = 3). Statistical significance was determined using a two-tailed Student’s *t*-test (*p* < 0.05).

To evaluate the effects of sex hormones and growth factors on cell viability, 3D co-cultured aggregates (Group 3 and Group 4) were analyzed using the Live/Dead Viability/Cytotoxicity Kit and a trypan blue staining assay. As shown in Figure 2A, most of the cells across both conditions remained viable, exhibiting strong green fluorescence regardless of hormone and growth factor supplementation. Furthermore, compared to the negative control, no red fluorescence was detected within the 3D aggregates. Quantitative assessment via trypan blue staining corroborated these observations, confirming high cell viability under both conditions with no statistically significant difference (Figure 2B; 91.0 ± 0.4% in Group 3 vs. 93.0 ± 1.65% in Group 4, n.s.). These results demonstrated that the addition of sex hormones and growth factors does not adversely affect cell viability of 3D co-cultured aggregates.

### 3.3. Effects of Sex Hormones and Growth Factors on Germ Cell Marker Gene Expression

**Figure 3:**
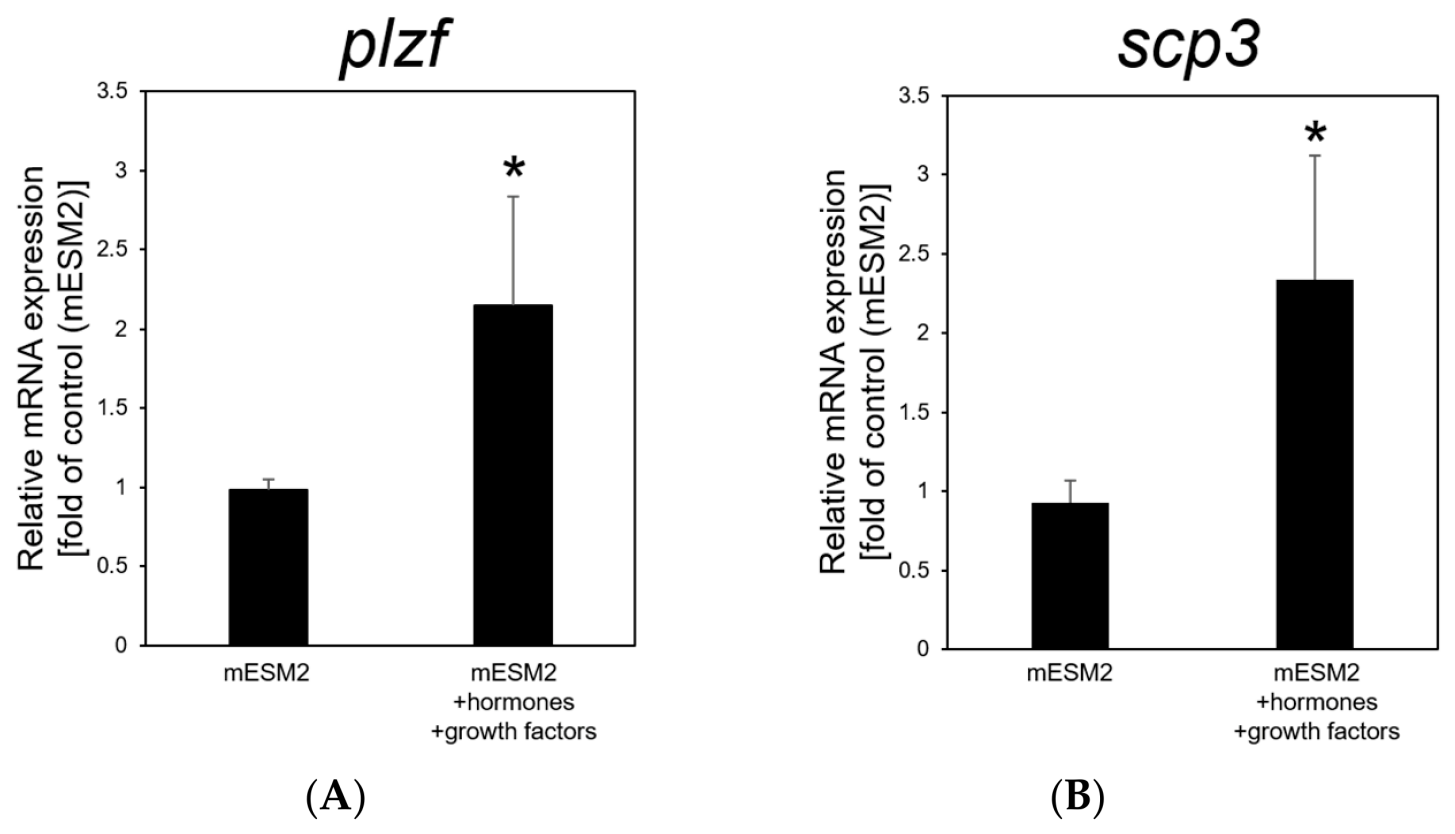
Effects of sex hormones and growth factors on germ cell marker expression in 3D co-cultured testicular aggregates. (**A**) Relative mRNA expression of *plzf*. (**B**) Relative mRNA expression of *scp3*. Data are presented as mean ± SD (n = 3). Asterisks (*) indicate a statistically significant difference. Statistical significance was determined using a two-tailed Student’s *t*-test (*p* < 0.05).

To evaluate the functional effects of sex hormones and growth factors, the transcript levels of *plzf* (a spermatogonial stem cell marker) and *scp3* (a meiotic germ cell marker) in 3D co-cultured aggregates were analyzed by qRT-PCR. As a result, supplementation with sex hormones and growth factors in mESM2 significantly upregulated the expression of both *plzf* and *scp3*, showing 2.15 ± 0.69-fold and 2.34 ± 0.79-fold increases, respectively, compared to the untreated control (Figure 3). These results demonstrate that sex hormone and growth factor supplementation play a critical role in enhancing both the maintenance and meiotic differentiation of spermatogonial stem cells within 3D co-cultured aggregates.

## 4. Discussion

In this study, OFT cell line was successfully applied to form 3D co-cultured testicular aggregates in *P*. *olivaceus*. Furthermore, the specific effects of sex hormone and growth factor supplementation on the viability and functionality of these 3D testicular aggregates were evaluated.

Previously, Edmonds and Woodruff demonstrated that primary testicular cells isolated from 5 and 12 dpp male mice self-assembled into testicular organoids within 24 h in non-adherent microwells, whereas primary cells from adult mice (8–16 weeks) failed to aggregate under identical conditions [19]. Notably, co-culturing adult testicular cells with immature murine testicular cells rescued this self-aggregation capacity, leading to the hypothesis that Sertoli cells are indispensable for testicular organoid assembly. Similarly, Alves-Lopes et al. suggested that the abundance of Sertoli cells in immature testes plays a pivotal role in driving organoid formation [20]. Consistent with these mammalian findings, primary testicular cells cultured alone in ULA plates failed to form aggregates within 24 h in the present study. In contrast, co-culturing primary testicular cells with the OFT cell line initiated rapid aggregation within 24 h. These results strongly demonstrate that the OFT cell line acts as a critical structural and functional feeder layer that drives and accelerates 3D aggregate assembly in *P. olivaceus*.

Compared to our previous study on marine medaka (*Oryzias dancena*), where testicular cells self-assembled without a specific feeder line [2], the *P*. *olivaceus* primary cells demonstrated a dependency on OFT co-culture for initial assembly. It highlights species-specific differences in somatic cell requirements and reinforces the necessity of the OFT cell line as an active somatic support.

Subsequent evaluation revealed that while supplementation with sex hormones (11-KT, E_2_, DHP, and hCG) and growth factors (EGF and IGF-1) did not adversely affect cell viability, it significantly upregulated the expression of both *plzf* and *scp3*. In teleosts, these factors play distinct roles in germ cell dynamics. For instance, 11-KT is essential for initiating meiotic entry in tilapia, whereas IGF-1 and hCG stimulate spermatogonial stem cell (SSC) proliferation [21]. Likewise, 11-KT treatment induced high expression of meiotic markers (*rec8*, *scp3*, and *dmc1*) and generated sperm-like cells in an orange-spotted grouper SSC line [8]. Regarding E_2_, several studies have highlighted its role in stimulating early germ cell proliferation, such as in cryptorchid mice [22] and via in vivo administration in the Japanese eel [23]. Additionally, DHP and its receptor are expressed during early spermatogenesis in Japanese eel, directly inducing meiotic entry [24]. EGF also serves as a key mitogenic factor widely utilized in mammalian SSC cultures, including rat [25], mouse [26], and human [27].

Based on these functional roles reported in prior studies, it can be speculated that the significant increase in *plzf* expression observed in our 3D testicular aggregates was primarily driven by the proliferative actions of E_2_, hCG, IGF-1, and EGF, whereas the upregulation of scp3 was induced by the meiotic effects of 11-KT and DHP. However, since a combined cocktail of hormones and growth factors was applied in the present study, the precise individual contribution and synergistic interplay of each factor remain to be elucidated. Systematic evaluations testing each factor independently will be required in future investigations to optimize the supplementation for long-term spermatogonial stem cells maintenance and differentiation.

It is important to note that the qRT-PCR in this study was performed on whole aggregates, which consist of a mixed population of primary testicular cells and OFT cells. Therefore, the observed increase in *plzf* and *scp3* normalized to total RNA might reflect a change in the relative proportion of germ cells rather than solely an increase in transcription rate per cell. Furthermore, the germ cell content was evaluated based on these two transcripts without the use of supplementary germ-cell markers or direct immunostaining. Additionally, since baseline gene expression levels in isolated cells (day 0) were not evaluated, the precise temporal dynamics of how germline-associated expression changes relative to the initial in vivo population remain an important subject for future investigation.

Furthermore, while the live/dead assay confirmed high overall cell viability throughout the intact 3D aggregates, detailed histological analyses, such as immunohistochemistry to determine the structural organization and cell type distribution, were not performed in this preliminary study. Investigating the cell–cell architecture and spatial compartmentalization within the 3D aggregate will be critical to fully understand how the OFT niche physically interacts with and supports germ cell progression.

Importantly, the dependency of germ cell organization on somatic support and the hormonal control of spermatogonial maintenance and meiotic entry are highly conserved mechanisms across vertebrates, extending well beyond teleosts. The interactions observed here mirror the critical Sertoli–germ cell dynamics required in mammalian and human in vitro spermatogenesis models. By demonstrating these evolutionarily conserved interactions in a marine teleost, our 3D OFT-supported system not only provides a scalable platform for aquaculture but also offers a comparative in vitro model. This broadens the understanding of fundamental germ cell–somatic cell interactions and functional niche reconstruction relevant to broader reproductive biology.

Taken together, the established 3D co-culture system using the OFT cell line provides an in vitro platform for promoting teleost testicular aggregate formation and enhancing germline gene expression, serving as a foundational tool for advanced reproductive biotechnology in aquaculture.

## 5. Conclusions

In conclusion, this study successfully established a novel 3D co-culture aggregate system for olive flounder (*Paralichthys olivaceus*) primary testicular cells utilizing the established OFT cell line. Our findings demonstrated that while primary testicular cells alone fail to self-assemble, co-culturing with OFT cells plays a critical role in promoting and accelerating robust 3D testicular aggregate formation and facilitating germ cell incorporation. Furthermore, supplementation with a cocktail of sex hormones (11-KT, DHP, E_2,_ hCG) and growth factors (EGF, IGF-1) maintained high cell viability while significantly upregulating the transcript levels of both the spermatogonial stem cell marker (*plzf*) and the meiotic germ cell marker (*scp3*) compared to un-supplemented aggregates. Overall, this 3D co-culture platform offers a valuable in vitro model for maintaining, proliferating, and differentiating teleost germline stem cells, providing a foundational tool for advanced reproductive biotechnology and germ cell preservation in aquaculture.

## Figures and Tables

**Table 1:** The information of primer sequences used in this study.

Gene		Primer Sequences (5′ > 3′)	Size (bp)	Accession No.
*plzf*	Forward	TCCTCTTCCACCGCAACAG	79	XM_020097052.1
	Reverse	GCATACTCCAAAATCTGCTGAAAA		
*scp3*	Forward	TGGCTACCGTCCGCAAGT	154	XM_020090502.1
	Reverse	CGATATGAACACGAACCAAATTAAGT		
*18s rRNA*	Forward	ATGGCCGTTCTTAGTTGGTG	218	EF126037.1
	Reverse	CACACGCTGATCCAGTCAGT		

## Data Availability

The datasets used or analyzed during the current study are available from the corresponding author upon reasonable request.

## References

[1] Lubzens E., Young G., Bobe J., Cerdá J. (2010). Oogenesis in teleosts: How fish eggs are formed. Gen. Comp. Endocrinol..

[2] Choi J.H., Ryu J.H., Gong S.P. (2023). Establishment and optimization of an aggregate culture system of testicular cells from marine medaka, *Oryzias dancena*. J. Mar. Sci. Eng..

[3] Yoshizaki G., Yazawa R. (2019). Application of surrogate broodstock technology in aquaculture. Fish. Sci..

[4] Iwasaki-Takahashi Y., Shikina S., Watanabe M., Banba A., Yagisawa M., Takahashi K., Fujihara R., Okabe T., Valdez D.M.J.R., Yamauchi A. (2020). Production of functional eggs and sperm from in vitro-expanded type A spermatogonia in rainbow trout. Commun. Biol..

[5] Hong Y., Liu T., Zhao H., Xu H., Wang W., Liu R., Chen T., Deng J., Gui J.F. (2004). Establishment of a normal medaka fish spermatogonial stem cell line capable of sperm production in vitro. Proc. Natl. Acad. Sci. USA.

[6] Sun X., Tao B., Wang Y., Hu W., Sun Y. (2022). Isolation and characterization of germline stem cells in the hermaphroditic fish *Monopterus albus*. Int. J. Mol. Sci..

[7] Chen X., Kan Y., Zhong Y., Jawad M., Wei W., Gu K., Gui L., Li M. (2022). Generation of a long-term-cultured Chinese hook snout carp spermatogonial stem cell line capable of sperm production in vitro. Biology.

[8] Zhong C., Tao Y., Liu M., Wu X., Yang Y., Wang T., Meng Z., Xu H., Liu X. (2022). Establishment of a spermatogonial stem-cell line with potential of models in a hermaphroditic fish, *Epinephelus coioides*. Cells.

[9] Tan L., Liu Q., He Y., Zhang J., Hou J., Ren Y., Ma W., Wang Q., Shao C. (2023). Establishment of a spermatogonial stem-cell line from tiger puffer fish. Animals.

[10] Kawasaki T., Saito K., Sakai C., Shinya M., Sakai N. (2012). Production of zebrafish offspring from cultured spermatogonial stem cells. Genes Cells.

[11] Wong T.T., Tesfamichael A., Collodi P. (2013). Production of zebrafish offspring from cultured female germline stem cells. PLoS ONE.

[12] Cho S.H., Kim K.T., Choi I.C., Jeon G.H., Kim D.S. (2012). Compensatory growth of grower olive flounder (Paralichthys olivaceus) with different feeding regime at sub-optimal temperature. Asian-Australas. J. Anim. Sci..

[13] Park J.W., Lee D.I., Jung H.S., Kim J., Yang H.R., Lee J.H. (2021). Estimation of genetic parameters and improvement of growth traits in selected olive flounder. Korean J. Fish. Aquat. Sci..

[14] Kim D.S., Jeong C.W., Lee Y.-D., Rho S. (1994). Triploidy induction in olive flounder (*Paralichthys olivaceus*). J. Aquac..

[15] Ko M.G., Jung H.S., Lee H.B., Kim D.S. (2016). Cytogenetic analysis of all-female triploid olive flounder for ploidy verification. Korean J. Fish. Aquat. Sci..

[16] Kim J., Cho J.Y., Kim J.W., Kim H.C., Noh J.K., Kim Y.O., Hwang H.K., Kim W.J., Yeo S.Y., An C.M. (2019). CRISPR/Cas9-mediated myostatin disruption enhances muscle mass in olive flounder. Aquaculture.

[17] Jo J.Y., Kim J.-W., Noh E.S., Kim Y.-O., Gong S.P., Kong H.J., Choi J.H. (2025). Establishment and characterization of OFT and OFO cell lines from olive flounder (*Paralichthys olivaceus*) for use as feeder cells. Biology.

[18] Livak K.J., Schmittgen T.D. (2001). Analysis of relative gene-expression data using real-time quantitative PCR and the 2[−ΔΔCT] method. Methods.

[19] Edmonds M.E., Woodruff T.K. (2020). Testicular organoid formation is a property of immature somatic cells that self-assemble and exhibit long-term hormone-responsive endocrine function. Biofabrication.

[20] Alves-Lopes J.P., Soder O., Stukenborg J.B. (2017). Testicular organoid generation by a novel in vitro three-layer gradient system. Biomaterials.

[21] Tokalov S.V., Gutzeit H.O. (2005). Spermatogenesis in testis primary cell cultures of the tilapia (*Oreochromis niloticus*). Dev. Dyn..

[22] Li Z.E., Li X.D., Zhang Q.S., Wang Y.C., Zhang M.X., Lu Y.J., Duan C.M., Yang X.Z., Feng L.X. (2007). 17 beta-estradiol stimulates proliferation of spermatogonia in experimental cryptochid mice. Asian J. Androl..

[23] Miura T., Miura C., Ohta T., Nader M.R., Todo T., Yamauchi K. (1999). Estradiol-17β stimulates the renewal of spermatogonial stem cells in males. Biochem. Biophys. Res. Commun..

[24] Miura T., Higuchi M., Ozaki Y., Ohta T., Miura C. (2006). Progestin is an essential factor for the initiation of the meiosis in spermatogenetic cells of the eel. Proc. Natl. Acad. Sci. USA.

[25] Wahab-Wahlgren A., Martinelle N., Holst M., Jahnukainen K., Parvinen M., Soder O. (2003). EGF stimulates rat spermatogonial DNA synthesis in seminiferous tuble segments in vitro. Mol. Cell Endocrinol..

[26] Azizi H., Skutella T., Shahverdi A. (2017). Generation of mouse spermatogonial stem-cell-colonies in a non-adherent culture. Cell J..

[27] Medrano J.V., Rombaut C., Simon C., Pellicer A., Goossens E. (2016). Human spermatogonial stem cells display limited proliferation in vitro under mouse spermatogonial stem cell culture conditions. Fertil. Steril..

